# Comparison between swallowing and chewing of garlic on levels of serum lipids, cyclosporine, creatinine and lipid peroxidation in Renal Transplant Recipients

**DOI:** 10.1186/1476-511X-4-11

**Published:** 2005-05-19

**Authors:** Abbas Jabbari, Hassan Argani, Amir Ghorbanihaghjo, Reza Mahdavi

**Affiliations:** 1 Clinical pharmacy laboratory, Drug Applied Research Center, Tabriz University of Medical Sciences, University Ave., Tabriz, Iran; 2 Clinical pharmacy laboratory, Drug Applied Research Center, Tabriz University of Medical Sciences, University Ave., Tabriz, Iran; 3 Biochemistry and Drug Metabolism Laboratory, Drug Applied Research Center, Tabriz University of Medical Sciences,, University Ave., Tabriz, Iran; 4 Nutrition Laboratory, Drug Applied Research Center, Tabriz University of Medical Sciences, University Ave., Tabriz, Iran

## Abstract

Abstract   Hyperlipidemia  and  increased  degree  of  oxidative  stress  are  among  the  important   risk  factors  for  Atherosclerosis  in  renal  transplant  recipients  (RTR).  The  Medical   treatment of hyperlipidemia in RTR because of drugs side effects has been problematic,   therefore  alternative  methods  such  as  using  of  Garlic  as  an  effective  material  in   cholesterol lowering and inhibition of LDL Oxidation has been noted. For evaluation of   garlic effect on RTR, 50 renal transplant patients with stable renal function were selected   and divided into 2 groups. They took one clove of garlic (1 gr) by chewing or swallowing   for  two months,  after  one month wash-out  period,  they  took  garlic  by  the  other  route.   Results indicated that although lipid profile, BUN, Cr, serum levels of cyclosporine and   diastolic blood pressure did not change, Systolic blood pressure decreased from138.2 to   132.8 mmHg (p=0.001) and Malondialdehyde (MDA) decreased from 2.4 to1.7 nmol/ml   (p=0.009)  by  swallowing  route,  Cholesterol  decreased  from  205.1  to  195.3  mg/dl   (p=0.03),  triglyceride  decreased  from  195.7  to 174.8 mg/dl  (p=0.008), MDA  decreased   from 2.5 to 1.6 nmol/ml (p=0.001), systolic blood pressure decreased from 137.5 to 129.8   mmHg (p=0.001), diastolic blood pressure decreased from 84.6 to 77.6 mmHg (p=0.001)   and  Cr  decreased  from  1.51  to  1.44 mg/dl  (p=0.03)  by  chewing  route  too.   However   HDL,  LDL  and  cyclosporine  serum  levels  had  no  significant  differences  by  both  of   swallowing and chewing routes.   We conclude that undamaged garlic (swallowed) had no lowering effect on lipid level of   serum. But Crushed  garlic  (chewed)  reduces  cholesterol,  triglyceride, MDA  and  blood   pressure. Additionally creatinine reduced without notable decrease in cyclosporine serum   levels may be due to cyclosporine nephrotoxicity ameliorating effect of garlic.

## Introduction

Cardiovascular disease (CVD) is the main cause of morbidity and mortality in renal transplant recipients [1] and it accounts for about 40% of deaths in this group of patients [2]. Several risk factors for CVD, such as lipid abnormalities and insulin resistance, may partly explain the accelerated development of atherosclerosis following renal transplantation [1]. In atherogenesis process, macrophages oxidate LDL and produce foam cells by OX-LDL that are characteristic for atherosclerosis [3]. Lipoprotein abnormalities are common in renal disease [4] these are reported in 50–80% of renal transplant recipients [5].

Pathogenesis of hyperlipidemia in Renal transplant recipients (RTR) is not fully understood, but dosage of steroid, consumption of cyclosporine, anti-hypertensive medication, rising of serum creatinine, proteinuria and diabetes mellitus have been considered [2].

There is increased degree of oxidative stress in RTR [6]. In these patients following hyperlipidemia and lipoprotein abnormalities, free radicals produced. Also immunosuppressive drugs can induce photosensitization reactions. These reactions lead to production of free radicals and aggravate lipid peroxidation [7].

The medical treatment of hyperlipidemia in transplant recipients is problematic [2]. Bile binding resins interfere with cyclosporine absorption and also lead to hyper triglyceridemia. Nicotinic acid causes hyperglycemia and rising of uric acid. Fibric acids cause myopathy, dyspepsia, gall bladder stones and rises of creatinine. Statins can induce hepatotoxicity, myopathy rhabdomyolysis (Especially if accompanied with cyclosporine) [2,4,5]. So alternative methods are deserved importance.

Garlic has been used for centuries as an herbal medicine in treating abscesses, cough, poisoning, parasites, worms, digestive and circulatory problems, snake bites [8] hemorrhoids, abdominal pain, loss of appetite and pneumonia [9]. Epidemiologic studies suggest that consumption of garlic may protect against carcinogenesis. In particular, the development of gastric and colorectal cancers seems to be prevented by alliums consumption [10,11]. Also garlic was known as an effective material in decreasing of blood pressure [12] and cholesterol [13,14] also can inhibit LDL oxidation [15-17], platelet aggregation and adhesion [18,19] and can increase Nitric oxide production [20]. Because of these beneficial effects of garlic, we decided to study the effect of garlic on lipid profile, lipid peroxidation and cyclosporine serum level and because of its irritant odor in chewing we chose two routes of swallowing and chewing for comparing of their efficacy.

## Method and materials

50 renal transplant recipients with stable renal function (based on serum Cr<1.8 mg/dl) were selected randomly (hyperlipidemic and normolipidemic) (Table 1)(see additional file 1, Table1, word). Patients had been transplanted more than 1 year and were treating with triple immunosuppressive regimen including of: cyclosporine, prednisolone and azathioprine / or mycophenolate mofetil. Some of patients were under hypolipemic agents and antihypertensive drugs. Drug regimen did not change in 2 months period before and during the study.

We divided patients into 2 groups randomly (A, B). Group A patients took one clove of raw aged garlic (1 gr) by swallowing and group B patients by chewing the same amount. At the start of study we checked their dietary regimen including of: intake of calorie, total fat (TF), saturated fatty acids (SFA), monounsaturated fatty acids (MUFA), polyunsaturated fatty acids (PUFA) and cholesterol. We checked also some clinical and Para clinical parameters including of: blood pressure, weight, triglyceride (TG), cholesterol (Chol), low density lipoprotein (LDL), high density lipoprotein (HDL), serum malondialdehyde (MDA), blood urea nitrogen (BUN), creatinine (Cr) and cyclosporine serum level. Patients took garlic for 2 months then we checked above parameters again. After one month wash-out period the patients took garlic by the other route (swallowing changed to chewing and vice versa) with the same condition (as a cross over design).

Lipid profiles (TG, Chol, LDL, HDL), BUN and Cr were measured by standard enzymatic method, MDA determined by colorimetric method using thiobarbituric acid reactions [21] and cyclosporine serum level measured by RIA.

Data were analyzed by paired sample t test and Non parametric 2 related sample test using SPSS11.5 program. A difference was considered statistically significant when the P value was <0.05.

## Results

In this study 2 patient (group A) because of heart burn, 1 patient (group A) because of bloating, 3 patients because of change in drug regimen were excluded (group B) 44 patients continued the study (22 patients in each group).

In patients who swallowed garlic, weight, intake of calorie, SFA, MUFA, PUFA had no significant differences but intake of TF and Chol were increased during the study as compared with the pre garlic period. In patients who chewed garlic, weight, intake of calorie did not change however intake of TF, SFA, MUFA, PUFA and Chol increased during the study (table 2) (see additional file 2, Table 2, word).

Comparison between results of Chewing and Swallowing of garlic indicate that there is significant differences in diastolic blood pressure (P = 0.016), triglyceride (P = 0.008) and cholesterol (P = 0.04) but not in systolic blood pressure (P = 0.187), HDL (P = 0.925), LDL (P = 0.354), MDA (P = 0.587), BUN (P = 0.657), Cr (P = 0.119) and cyclosporine serum level (P = 0.155). Other data are provided in table3 (see additional file 3, Table3, word).

## Conclusion

Several factors after transplantation produce hyperlipidemia include: weight gain and increase body fat mass due to appetite improvement [22].

Lopes et al reported that moderate energy restriction of about 30% and reducing fat in diet decreased cholesterol and LDL [23] however in our study dietary intake not only did not decreased but it increased significantly.

Adler and holub showed that LDL were reduced (14.2%) and total cholesterol were significantly lower (11.5%) with taking 900 mg garlic/day for 12 weeks in hypercholesterolemic men [24].

Tohidi and Rahbani showed that taking 1200 mg garlic powder for 4 weeks reduced total cholesterol (9%), triglyceride (11%), LDL (15%), systolic blood pressure (3%) and diastolic blood pressure (2%) [15].

Steiner et al with giving 7.2 gr aged garlic extract (AGE) for 4 weeks indicated reduction in cholesterol (6.1%), LDL (4%), systolic and diastolic blood pressure (5.5%) [14].

Issacsohn et al reported that taking 900 mg garlic powder for 12 week did not change in lipid profile [8].

Mader showed that taking 800 mg garlic powder for 4 months reduced cholesterol (9%) and triglyceride (15%) [25].

Lash et al reported that taking garlic tablets at a dose of 680 mg two times a day for 6, 12 weeks decreased LDL (6%) and total cholesterol (4%) significantly in hypercholesterolemic renal transplant patients [2].

Brinker mentioned that Effectiveness might be decreased by garlic's ability to induce metabolism and decrease levels of drugs like cyclosporine which are substrates of cytochrome P450 3A4. It can potentially cause transplant rejection [26].

Blech et al showed progression of atherosclerosis is relevant with oxidative stress and indicated with measurement of MDA [27].

MDA is important marker of lipid peroxidation [28] and reported that serum MDA decreased by antioxidants consumption [29].

In this study after chewing of garlic, systolic and diastolic blood pressure decreased 5%, 8%, like as Steiner and Tohidi studies. Also cholesterol and triglyceride reduced 4%, 10%, according to Mader and Tohidi studies but less than these studies. Decreased Cholesterol in this study was like as Lash study however he did not report triglyceride reduction after garlic consumption. HDL did not change in all studies. Cyclosporine serum level decreased not significantly in our study and as we known it is the first study which demonstrated the effect of garlic on cyclosporine level in RTR.

Ingestion of garlic by chewing (or crushed garlic) can reduce cholesterol, triglyceride, MDA, systolic and diastolic blood pressure even in the presence of increasing fat intake. But undamaged garlic (swallowed) had no significant effect on serum lipids (TG, Chol, LDL and HDL), diastolic blood pressure, and BUN, Cr and cyclosporine serum level. Our hypothesis is that it is because of inability of Alliin to convert to Allicin. So the specific garlic odor is a hallmark of releasing of Allicin.

Acute cyclosporine nephrotoxicity is predominantly functional in that it produces no particular histological features. It is probably the result of renal arteriolar constriction, a feature that has been demonstrated in animals. In chronic cyclosporine nephrotoxicity the principal injury is to the small arterioles where there is vacuolation of smooth muscle and endothelial cells [30].

Reducing creatinine without notable decrease in cyclosporine serum level by chewing of garlic may be cyclosporine nephrotoxicity protecting effect of garlic as its effect on gentamycin nephrotoxicity due to its antioxidant effect [31] or calcium channel blockers like effects [32] or nitric oxide increasing vasodilatations [20].

Additional studies are necessary in order to investigate effect of garlic on cyclosporine serum levels, nephrotoxicity and serum creatinine.

In conclusion we found that crushed garlic (chewed) reduced total cholesterol, triglyceride, MDA (lipid peroxidation) and blood pressure that have important role in cardiovascular disease. Therefore garlic consumption can prevent this disease.

## Supplementary Material

Additional File 1 Table 1 Click here for file

Additional File 2 Table 2 Click here for file

Additional File 3 Table 3 Click here for file
