# Supplementary material for: Comparison between swallowing and chewing of garlic on levels of serum lipids, cyclosporine, creatinine and lipid peroxidation in Renal Transplant Recipients
Source: Lipids Health Dis. 2005 May 19;4:11. doi: 10.1186/1476-511X-4-11 (PMC1173136; doi:10.1186/1476-511X-4-11)
Supplement: Additional File 1 — Table 1 [file 1476-511X-4-11-S1.doc]

Additional file 1

Table 1

File type: Word

Table 1: Baseline data

|  | Male | Female | Total |
| --- | --- | --- | --- |
| Age (month) | 40.8±14.4 | 37.8±12.3 | 39.4±13.3 |
| Months after transplant | 46.9±41.8 | 57±42.4 | 51.5±41.6 |
| BMI (Kg/m^2^) | 25.6±3.7 | 26.7±3.1 | 26.1±3.4 |
| Waist/hip (whr) | 0.90±0.06 | 0.84±0.05 | 0.87±0.06 |
