# Supplementary material for: Comparison between swallowing and chewing of garlic on levels of serum lipids, cyclosporine, creatinine and lipid peroxidation in Renal Transplant Recipients
Source: Lipids Health Dis. 2005 May 19;4:11. doi: 10.1186/1476-511X-4-11 (PMC1173136; doi:10.1186/1476-511X-4-11)
Supplement: Additional File 2 — Table 2 [file 1476-511X-4-11-S2.doc]

Additional file 2

Table 2

File type: Word

Table 2: weight and dietary regimen

|  |  | Swallowing | | |  |  | Chewing | | |  |
| --- | --- | --- | --- | --- | --- | --- | --- | --- | --- | --- |
|  | Before | | After | P value | | Before | | After | P value | |
| Weight (kg) | 68±12.3 | | 68±11.9 | 0.863 | | 67.9±11.6 | | 67.9±11.8 | 0.964 | |
| Energy (kcal) | 1842±1163 | | 1817±1002 | 0.765 | | 1675±786 | | 1600±1175 | 0.341 | |
| TF (gr) | 52±53 | | 56±46 | 0.005 | | 35±32 | | 42±30 | 0.001 | |
| SFA (gr) | 10±9 | | 10.2±8 | 0.432 | | 8.7±7 | | 9.9±9 | 0.005 | |
| MUFA (gr) | 5.7±7 | | 6.5±14 | 0.168 | | 4.5±8 | | 5.6±7 | 0.001 | |
| PUFA (gr) | 1.5±3.5 | | 3.5±12 | 0.092 | | 1.3±3 | | 1.6±2.8 | 0.001 | |
| Chol (mg) | 137±226 | | 154±272 | 0.042 | | 110±267 | | 153±159 | 0.035 | |
