# Supplementary material for: Comparison between swallowing and chewing of garlic on levels of serum lipids, cyclosporine, creatinine and lipid peroxidation in Renal Transplant Recipients
Source: Lipids Health Dis. 2005 May 19;4:11. doi: 10.1186/1476-511X-4-11 (PMC1173136; doi:10.1186/1476-511X-4-11)
Supplement: Additional File 3 — Table 3 [file 1476-511X-4-11-S3.doc]

Additional file 3

Table 3

File type: Word

Table 3: Total results

|  |  | Swallowing | | |  |  | Chewing | | |  |
| --- | --- | --- | --- | --- | --- | --- | --- | --- | --- | --- |
|  | Before | | After | P value | | Before | | After | P value | |
| TG (mg/dl) | 180.5±100.8 | | 189.3±91.7 | 0.27 | | 195.7±100.3 | | 174.8±91.9 | 0.008 | |
| Chol (mg/dl) | 205.1±40.5 | | 205±36.2 | 0.97 | | 205.1±36.5 | | 195.3±34.2 | 0.03 | |
| LDL (mg/dl) | 115.8±32.7 | | 115±28.8 | 0.85 | | 108.3±35.9 | | 108±32 | 0.96 | |
| HDL (mg/dl) | 54.7±11.7 | | 53.3±10.3 | 0.48 | | 55.1±13.5 | | 53.9±8.6 | 0.54 | |
| MDA (nmol/ml) | 2.4±1.3 | | 1.7±0.6 | 0.009 | | 2.5±1 | | 1.6±0.6 | 0.001 | |
| SBP (mmHg) | 138.2±23.6 | | 132.8±23.4 | 0.001 | | 137.5±21.7 | | 129.8±25.5 | 0.001 | |
| DBP(mmHg) | 83±10.5 | | 80.7±9.7 | 0.1 | | 84.6±9.5 | | 77.6±9.7 | 0.001 | |
| BUN (mg/dl) | 22±6.2 | | 22±9.1 | 0.95 | | 22.4±6.8 | | 22.3±6.9 | 0.85 | |
| Cr (mg/dl) | 1.45±0.33 | | 1.46±0.34 | 0.87 | | 1.51±0.30 | | 1.44±0.31 | 0.03 | |
| Cycl.lev (ng/ml) | 209.1±108.9 | | 223.4±90.6 | 0.36 | | 246.8±103.8 | | 223.3±95.3 | 0.16 | |

Data were analyzed by paired sample t test
